# Serum bilirubin to fetuin-A ratio as a prognostic biomarker in critically ill patients with sepsis

**DOI:** 10.1016/j.metop.2021.100094

**Published:** 2021-05-01

**Authors:** Irene Karampela, Maria Dalamaga

**Affiliations:** a Second Department of Critical Care, Attikon General University Hospital, Medical School, National and Kapodistrian University of Athens, Chaidari, Greece; b Department of Biological Chemistry, Medical School, National and Kapodistrian University of Athens, Athens, Greece

**Keywords:** Bilirubin, Critically ill, Fetuin-A, Mortality, Sepsis, Severity, Shock, APACHE, Acute Physiology and Chronic Health Evaluation, AUC, area under the curve, B/F ratio, serum bilirubin to fetuin-A ratio, CI, confidence interval, IQR, interquartile range, ROC, Receiver Operating Characteristic, SOFA, Sequential Organ Failure Assessment

## Abstract

Liver dysfunction during sepsis is associated with increased bilirubin and decreased fetuin-A, a major hepatokine. We aimed to explore the association of bilirubin to fetuin-A (B/F) ratio early in sepsis with severity and outcome in critically ill patients. Based on a previous prospective study, we analyzed data of 90 critically ill patients (52 males, age: 65 ± 15 years, APACHE II: 24 ± 7 and SOFA: 10 ± 3) with sepsis. Bilirubin and fetuin-A increased during the first week of sepsis, (median (IQR) 0.45 (0.32–1) vs 0.55 (0.29–0.78) mg/dL, p = 0.03 and 302 (248–336) vs 358 (307–399) μg/mL, p < 0.001, respectively) while the B/F ratio did not change significantly. However, the B/F ratio at baseline and one week later was significantly higher in patients with septic shock (N = 38) and nonsurvivors (N = 28) compared to patients with sepsis (N = 52) and survivors (N = 62), respectively. The B/F ratio was positively associated with severity scores and outperformed bilirubin as a predictor of mortality in ROC curve analysis (AUC 0.78 (0.69–0.88), p < 0.001 and 0.69 (0.57–0.8), p = 0.003 respectively). The B/F ratio may be a promising sepsis biomarker with possible predictive value in critically ill patients.

Sepsis, a life-threatening organ dysfunction due to infection, constitutes an important cause of death with a significant morbidity and mortality burden globally [1]. Due to its systemic nature, many organ systems may be involved. Liver function is affected in sepsis, manifesting as derangement of serum levels of many proteins synthesized in the liver. Bilirubin has been shown to increase during sepsis, serving as a useful biomarker with predictive value [2,3]. It is also included in clinical prediction tools such as the Sequential Organ Failure Assessment (SOFA) score that classifies severity and predicts outcome in critically ill patients [4]. Fetuin-A, also known as α2-HS-glycoprotein, is a functional protein mainly synthesized in the liver (hepatokine) exerting multiple actions involved in metabolic, immune and inflammatory processes [5,6]. Fetuin-A is an acute phase protein, since its secretion is variably altered in response to acute stimuli such as injury and sepsis [7].

In a previous study, we prospectively enrolled 102 critically ill patients and investigated serum fetuin-A at sepsis onset and one week later [8]. We found that serum fetuin-A was decreased early in sepsis in the majority of patients and recovered one week later, mainly in survivors. We also demonstrated that lower fetuin-A levels at enrollment and one week after as well as lower kinetics of fetuin-A during the first week from sepsis onset independently predicted 28-day mortality in critically ill patients with sepsis [8]. We further showed that fetuin-A to adiponectin ratio is independently associated with 28-day mortality [9].

Since hepatic secretion of both bilirubin and fetuin-A is affected during sepsis, but in opposite directions (hyperbilirubinemia and hypofetuinemia), we aimed to explore the association of serum bilirubin to fetuin-A (B/F) ratio early in sepsis with severity and outcome. Based on our previous study, we analyzed data from 90 critically ill patients (52 males, age 65 ± 15 years) with incident sepsis, at sepsis onset and one week later. Diagnosis of sepsis and septic shock was based on SEPSIS-3 criteria [10]. Patients with liver disease and those receiving total parenteral nutrition were excluded from the study. The study was approved by the Scientific and Ethical Committee of the hospital, and informed consent was given by study participants or their next of kin. The severity of critical illness was assessed by the Acute Physiology and Chronic Health Evaluation II score (APACHE II, 24 ± 7) and by the SOFA score (10 ± 3). B/F ratio was calculated using the formula 1000 x (Bilirubin/Fetuin-A) where both bilirubin and fetuin were expressed in μg/mL. The primary outcome was mortality rate at 28 days after enrollment.

Fifty two patients (58%) presented with sepsis and 38 patients (42%) with septic shock at enrollment. Twenty eight patients (31%) died within 28 days from enrollment. Serum bilirubin and fetuin-A increased one week after sepsis onset compared to baseline (median (IQR) 0.45 (0.32–1) mg/dL vs 0.55 (0.29–0.78) mg/dL, p = 0.03 and 302 (248–336) μg/mL vs 358 (307–399) μg/mL, p < 0.001, respectively). The B/F ratio did not change significantly during the first week of sepsis (median (IQR) 18 (9–34) vs 14 (7–29), p = 0.47). However, patients presenting with septic shock had significantly higher B/F ratio and lower fetuin-A both at enrollment and one week after, compared to patients presented with sepsis (B/F ratio, median (IQR): Day 1, 28 (10–48) vs 14 (9–23), p = 0.009; Day 7, 26 (11–40) vs 10 (6–20), p < 0.001; and fetuin-A, median (IQR): Day 1, 275 (209–333) vs 316 (285–348), p < 0.001; Day 7, 310 (230–357) vs 383 (342–431), p < 0.001). Of note, baseline bilirubin did not significantly differ between patients with sepsis and septic shock (median (IQR): 0.4 (0.32–0.72) vs 0.62 (0.34–1.49), p = 0.06). Additionally, nonsurvivors had also higher B/F ratio, higher bilirubin and lower fetuin-A both at enrollment and one week after, compared to survivors (B/F ratio: Day 1, 30 (18–48) vs 12 (8–25), p < 0.001; Day 7, 28 (21–39) vs 10 (7–16), p < 0.001; bilirubin: Day 1, 0.66 (0.4–1.49) vs 0.4 (0.3–0.78), p < 0.01; Day 7, 0.69 (0.6–1.19) vs 0.49 (0.26–0.71), p < 0.001; fetuin-A: Day 1, 214 (207–2300) vs 320 (286–342), p < 0.001; Day 7, 253 (214–310) vs 380 (342–431), p < 0.001). Furthermore, baseline B/F ratio presented a significant positive association with APACHE II and SOFA scores (r = 0.259, p = 0.01 and r = 0.286, p = 0.01 respectively). It was also significantly correlated with white blood cells (r = 0.246, p = 0.02), platelets (r = −0.219, p = 0.03) and procalcitonin (r = 0.247, p = 0.01) but not C-reactive protein. Receiver operating characteristic (ROC) analysis showed that baseline B/F ratio was a better predictor of 28-day mortality than bilirubin alone (Fig. 1) [11].

**Fig. 1:**
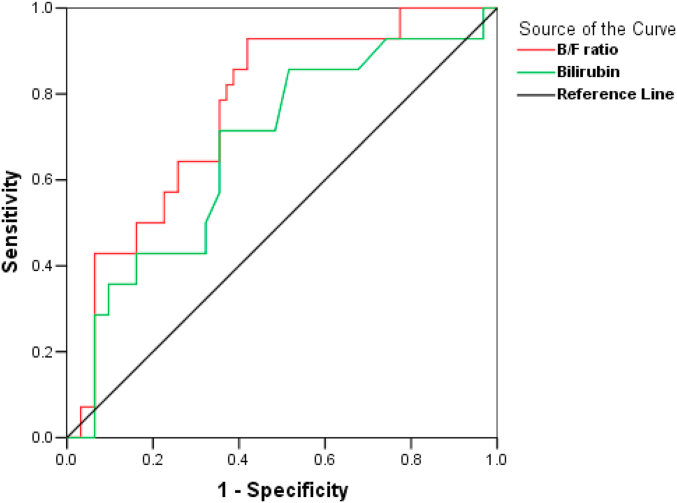
Receiver operating characteristic (ROC) analysis of bilirubin/fetuin-A (B/F) ratio and bilirubin in 90 critically ill patients at sepsis onset: B/F ratio AUC (area under the curve), 0.78 (95% CI, 0.69–0.88), p < 0.001; bilirubin AUC, 0.69 (95% CI, 0.57–0.8), p = 0.003.

Intrahepatic cholestasis, expressed as hyperbilirubinemia, is a well-known complication of sepsis, caused by inflammatory mediators and cell infiltrates in the liver, which suppresses the hepatobiliary transport systems leading to decreased bilirubin clearance [12,13]. Thus, bilirubin is a widely accepted biomarker of liver dysfunction in sepsis used in the SOFA score, which has been validated as a prognostic tool in critically ill patients [4]. Bilirubin has also been shown to exert anti-oxidant and anti-inflammatory properties, being a potential treatment for sepsis, as this has recently been shown in experimental sepsis in animal studies [14]. This finding suggests that at least mild hyperbilirubinemia may represent an adaptive response to sepsis [2]. Moreover, fetuin-A may act as an anti-inflammatory acute phase protein with predictive value, as previously shown [8]. The increased B/F ratio that we found in this study in more severely affected patients and in nonsurvivors may be explained by the severity of liver dysfunction (expressed by a higher serum bilirubin) along with the attenuated anti-inflammatory hepatic response (expressed by lower serum fetuin-A).

In conclusion, we demonstrated for the first time that the B/F ratio, which is a net number, is significantly higher in patients with septic shock compared to patients with sepsis, and in nonsurvivors compared to survivors, both at sepsis onset and one week later. We also showed that the B/F ratio is associated with the severity of sepsis. Interestingly, the B/F ratio predicts 28-day mortality in our cohort of septic critically ill patients better than bilirubin. Our study highlights a promising novel biomarker of sepsis in critically ill patients. Further prospective studies are needed to explore the possible predictive value of this index in larger populations of critically ill patients.

## Contributions of authors

Both authors have contributed equally to the conception of the idea, the design of the study, the literature search, analysis, writing, editing and revision of the manuscript.

## Funding source

None.

## Declaration of competing interest

The authors declare that they have no conflicts of interest.
